# The relationship between hematological parameters and coronary angiographic lesions

**DOI:** 10.3389/fcvm.2025.1589121

**Published:** 2025-08-21

**Authors:** Thadzia Maria de Brito Ramos, José Gildo de Moura Monteiro Júnior, Veridiana Câmara Furtado, Dário Celestino Sobral Filho

**Affiliations:** Pernambuco Cardiac Emergency Hospital, University of Pernambuco (PROCAPE, UPE), Recife, Brazil

**Keywords:** hematological parameters, nucleated red blood cells, neutrophil to lymphocyte ratio, mean platelet volume, coronary angiographic lesions, SYNTAX score, prognostic

## Abstract

Atherosclerosis is the most important etiology of acute myocardial infarction, which is considered an inflammatory disease with specific cellular and molecular responses. Recent research has linked hematological variables as biomarkers of the severity of coronary artery disease. Studies suggest that nucleated red blood cells (NRBCs), neutrophil to lymphocyte ratio (NLR), and mean platelet volume (MPV) may serve as components of a laboratory model or hematological scoring system for in-hospital surveillance. Atherosclerotic plaques can be graded using scoring systems, such as the SYNTAX score which is used to evaluate the complexity of coronary artery disease. However, there is an open field for research to explain the complex inflammatory mechanism of these plaques. Research has shown that inflammatory processes, such as those seen in coronary atherosclerotic disease, stimulate the bone marrow to release young and immature cells into the systemic circulation, which actively aggregate and, consequently, form thrombotic plaques. This mini review article aims to demonstrate the relationship between hematological parameters and coronary angiographic findings as potential in-hospital prognostic tools for patients with acute myocardial infarction. This expressive relationship between these hematological biomarkers and coronary atherosclerotic plaques may be a target, in addition to prognostic scores, of future therapeutic interventions.

## 1 Introduction

Atherosclerosis is mainly a systemic inflammatory disease, and inflammation plays an important role in the pathophysiology of acute coronary syndrome (1). The inflammatory and hypoxemic etiology of cardiovascular diseases stimulates the bone marrow, leading to an increased production of certain hematological cells or determining the appearance of immature cells (2). Previous works have shown hematological parameters as potential tools for in-hospital follow-up and prognosis of patients with acute myocardial infarction (AMI) (1–3).

**Figure 1:**
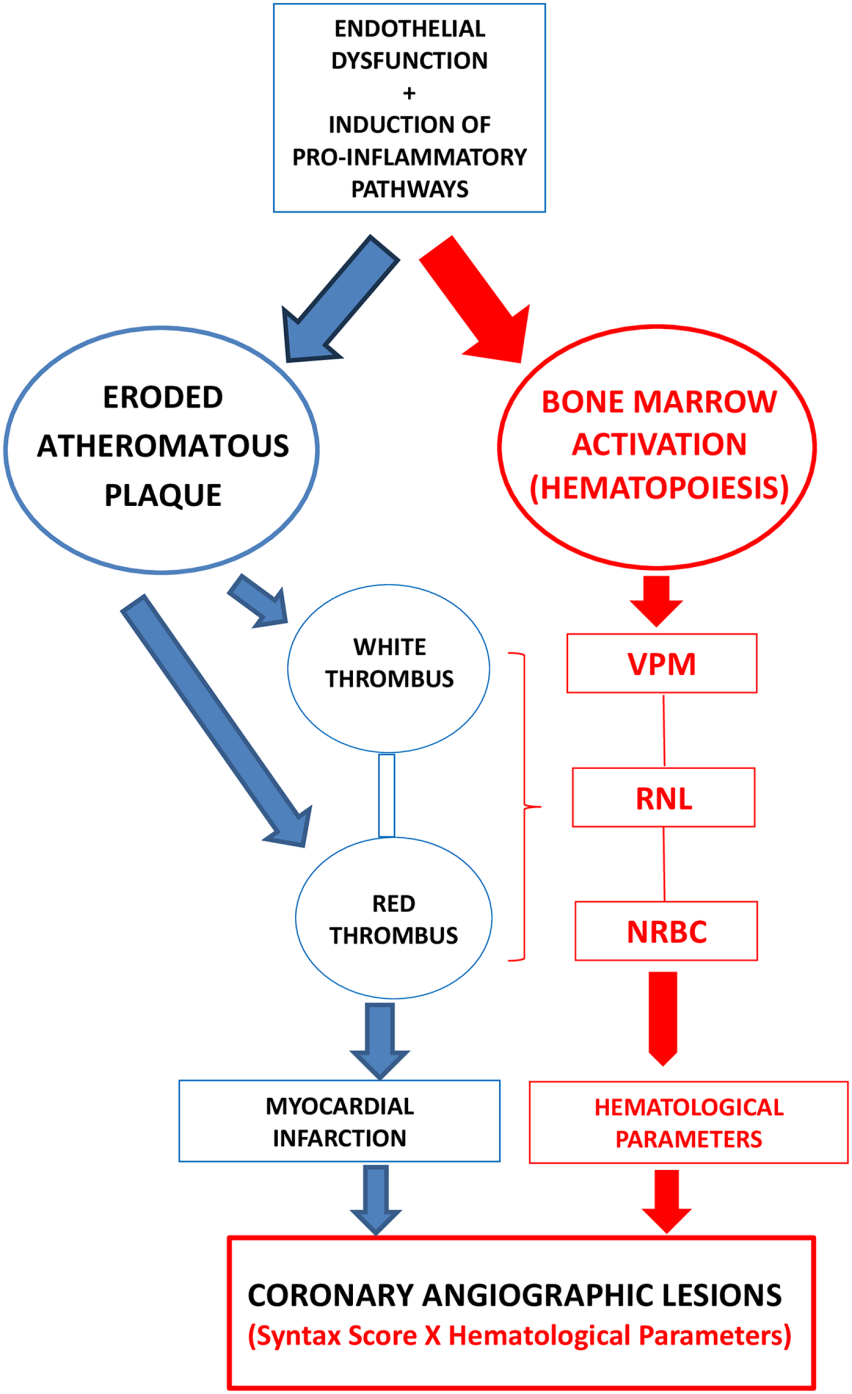
The relationship between atherosclerotic plaque and hematological parameters.

In this approach, all major blood count variables were represented: erythroblasts or nucleated red blood cell (NRBC) (red blood cells), neutrophil to lymphocyte ratio (NLR) (white blood cells), and mean platelet volume (MPV) (platelets) to evaluate the role relationship with coronary angiographic lesions (SYNTAX score) (Figure 1).

The SYNTAX score is used to verify the extent and severity of coronary artery disease (CAD) and has proven to be a good long-term prognostic marker in different scenarios of coronary artery disease, including patients with acute coronary syndromes (ACS) (4).

The relationship between hematological parameters and anatomical lesions provides new possibilities for hospital monitoring and prognosis of patients with AMI.

## 2 Hematological parameters

Atherosclerosis is a chronic inflammatory disease involving immune system activation that leads to endothelial dysfunction (5). It is mediated by lipids that induce numerous pro-inflammatory pathways and, together with bone marrow activation, induce myeloid changes, such as synthesis of nucleated red blood cells (NRBCs) and increased neutrophil to lymphocyte ratio (NLR) and mean platelet volume (MPV) in systemic circulation, further contributing to immune cell mobilization and plaque progression (2, 6). Thrombosis superimposed on an eroded or ruptured atherosclerotic plaque is responsible for life-threatening conditions such as acute coronary syndrome and stroke (7). The red thrombus is characterized by a lipid core, plaque rupture, necrotic core, neovascularization, intraplaque hemorrhage, and obstruction (8). Both types of thrombi include platelets and fibrin (9).

Bone marrow produces blood cells, a process called hematopoiesis, which is responsible for the production of red blood cells, leukocytes, and platelets. A single progenitor cell called a stem cell gives rise to all these cells when stimulated by erythropoietin, a glycoprotein produced in 90% of the kidneys, the rest in the liver. Blood exposure to low oxygen concentrations over a long time results in differentiation and increased production of red blood cells (3, 10). Research has shown that hypoxemia and infection trigger differentiation processes into distinct hematological cell lineages (10, 11).

### 2.1 Nucleated red blood cells

Nucleated red blood cells (NRBCs) are immature erythrocyte cells found in the bone marrow, normally in the peripheral blood of newborns for a few days, but not in adults. Its continuity in the peripheral circulation for children and adults is related to severe hypoxemia and/or infection when hematological diseases, cancer, congestive heart failure, acute and chronic anemias are excluded (12, 13). The presence of NRBCs in the peripheral circulation is associated with high concentrations of erythropoietin, interleukin-3, and interleukin-6 and a worse prognosis (12). Several studies suggest that the detection of NRBCs in hospitalized patients indicates an increased risk of mortality (14).

### 2.2 Neutrophil to lymphocyte ratio

The neutrophil to lymphocyte ratio (NLR) is composed of two independent markers of inflammation (15). Lymphocytes play an important role in the inflammatory response. Lymphopenia has been linked to progressive atherosclerosis in clinical studies (16). Research has shown an association between NLR and the severity and extent of coronary disease (17, 18). However, studies have disclosed a strong predictor of short- and long-term mortality in stable and unstable coronary diseases. Azab et al. (19) demonstrated that patients with non-ST-segment elevation myocardial infarction (NSTEMI) with NLR > 4.7 have a mortality rate of 29.8% compared with those with NLR < 3, with a mortality rate of 8.4% (*p* < 0.001).

Studies have shown that higher NLRs are related to a higher SYNTAX score in patients with non-ST-segment elevation acute coronary syndrome and higher numbers of involved coronaries (20, 21). In patients with unstable angina pectoris, NLR proved to be an independent predictor of cardiovascular mortality (22). Kaplangoray et al. (23) published that coronary thrombotic burden correlates with the neutrophil to lymphocyte ratio (*r* = 0.335, *p* < 0.001) and SYNTAX score (*r* = 0.351, *p* = 0.001).

Individuals with drug-eluting stent restenosis (ISR) are more likely to experience serious adverse cardiac events. Patients with higher NLR had higher SYNTAX scores II and greater proportions of vulnerable components of atherosclerotic plaques, demonstrating that NLR is a risk factor for in-stent restenosis (ISR) (24).

### 2.3 Mean platelet volume

The mean platelet volume (MPV) represents large platelets, which are metabolically and enzymatically more active than small platelets, with an important role in the extension of intravascular thrombus (25). Uysal et al. (26) showed in their study that the value of the MPV above 10.4 is a predictor of severe atherosclerosis with a sensitivity of 39% and specificity of 90% (ROC curve: 0.631, 95% CI: 0.549–0.708, *p* = 0.003). Therefore, MPV has been appointed as an independent risk factor for acute myocardial infarction in patients with coronary artery disease (27, 28). Studies have shown a positive correlation between VPM and SYNTAX score (29, 30).

### 2.4 Hematological score

The hematological score is composed of nucleated red blood cells (NRBC), mean platelet volume (MPV), and neutrophil to lymphocyte ratio (NLR), which represent the constituents of the blood count and whose changes are associated with all causes of hypoxemia and inflammation during hospitalization of patients with acute myocardial infarction. This scoring system was developed to monitor these variables, serving as a prognostic parameter. This hematological score, on a scale ranging from 0 to 49, has been shown to be associated with an increased risk of mortality (sensitivity of 89.1%, specificity 67.2%, negative predictive value of 97.9%, positive predictive value of 26.8% and area under the ROC curve of 86.8%). This work was published in 2020 by our research group, but still needs validation (2).

## 3 Angiographic lesions

The coronary angiographic lesions are classified by considering a scoring system, which is a tool for grading the complexity of coronary artery disease (31, 32). It specifies information on location, characteristics, tortuosity, and other factors for each lesion, which allowed the online calculator and a Kaplan–Meier curve to show what the cumulative event rate would have been for a patient at a similar risk level in the SYNTAX trial (33, 34). This score classifies coronary lesions into low SYNTAX score (0–22 points), intermediate (23–32 points), and high (≥33 points). Patients with low scores are ideal for percutaneous coronary intervention (PCI), while those with an intermediate or high SYNTAX score are preferentially eligible for surgery (34). Özmen et al. (35) demonstrated a significant relationship between the SYNTAX score and patients with diabetes mellitus.

### 3.1 SYNTAX score

The SYNTAX score originated from the work published by Patrick Serruys's group in 2009, which compared surgical revascularization with angioplasty using the first-generation *Taxus* drug-eluting stent (Boston Scientific, USA) in patients with a multi-arterial lesion pattern. The study was non-inferiority, and what was seen is that the primary outcome (death from any cause, stroke, myocardial infarction, or repeated revascularization) was more common in the angioplasty group (17.8% × 12.4%, *p* = 0.002). Mortality and infarction were similar in 1 year, and the surgical group experienced a higher incidence of stroke. Therefore, the SYNTAX score is an angiographic severity score of coronary lesions, assessing the difficulty and chance of success of the percutaneous intervention (PCI) (31).

### 3.2 SYNTAX score II

However, a second version (SYNTAX score II) was shown by Bo Xu's group in 2014, which consisted of adding clinical variables, which enlarged its performance in predicting mortality. The study examined 1,528 patients who underwent angioplasty of the unprotected left main coronary artery. The primary outcome was the ability of SYNTAX score II to predict mortality. Patients were divided into three tertiles: ≤21 points with 508 patients, >21 and ≤28 points with 480 patients, and >28 points with 540 patients. Those in the highest tertile were older, were more likely to be female, had a higher prevalence of prior heart attack and stroke, lower ejection fraction, reduced creatinine clearance, and more extensive coronary disease with complex lesions involving two or three vessels. At the 4-year follow-up, the rates of death, cardiac death, myocardial infarction, and target vessel revascularization were 4.4%, 5%, 7.5%, and 9.5%, respectively, which were significantly higher in the highest and intermediate tertiles. In the multivariate analysis, the SYNTAX score II was associated with an increased mortality rate (HR = 1.76, 95% CI: 1.1–2.82, *p* = 0.02) in patients receiving angioplasty in the unprotected main left coronary artery. The results suggest that SYNTAX score II has greater power to predict mortality compared with the classic SYNTAX angiographic score (36).

## 4 Hematological parameters and angiographic lesions

The relationship between hematological parameters and angiographic lesions is quite evident in studies published in the literature, especially the neutrophil to lymphocyte ratio and mean platelet volume (Table 1). However, there are still no studies evaluating the relationship between erythroblasts and angiographic lesions.

**Table 1:** Hematological parameters and SYNTAX score in clinical studies.

Clinical studies	SYNTAX score
Author (year)	Results
Neutrophil to lymphocyte ratio (NLR)	
Rostami et al. (2021)	After adjustments for age and sex, the NLR and the SII were independent predictors of postprocedural no reflow. However, the NLR and the SII are not predictors of the SYNTAX score and the preprocedural TIMI flow grade.
Maleki et al. (2021)	Higher NRL was significantly associated with higher SYNTAX score (*β* = 0,162, *p* = 0,021).
Li et al. (2023)	The level of NLR, PLR, and hsCAR and SII in patients with high SYNTAX score are significantly higher than those in patients with low SYNTAX score.
Tanındı et al. (2023)	Cutoff NLR to predict moderate to severe CAD according to SYNTAX score was 2.26, with 72% sensitivity and 71% specificity (AUC: 0.772, 95% CI: 0.679–0.865, *p* < 0.001).
Li et al. (2022)	NLR was positive and linearly correlated with SYNTAX score (*r* = 0.270).
Pan et al. (2022)	The NLR correlated with the Gensini and SYNTAX scores in male CAD patients (both *p* < 0.001).
Kahraman et al. (2021)	NLR with a cutoff value of 2.59 had good predictive value for increased rSS (area under the curve = 0.707, 95% CI: 0.661–0.752, *p* < 0.001). In conclusion, higher NLR was an independent predictor of increased rSS in patients with STEMI.
Mean platelet volume (MPV)	
Emre et al. (2020)	The rSS (*p* = 0:01) value of the high WMR group was higher than that of the low WMR group. A higher WMR value on admission was associated with worse outcomes in patients with P-PCI.
Vogiatzis et al. (2019)	MPV was significantly correlated to SYNTAX score (*r* = 0.658, *p* < 0.001) and was found to be an independent predictor factor of MACE with HR = 6.8 (95% CI: 1.46–33.36). The cutoff value of MPV was 7.5 with a sensitivity of 98% and a specificity of 30.8%.
Jiang et al. (2019)	Receiver operating characteristic analysis showed a good diagnostic value for MPV at predicting long-term cardiac mortality (area under the curve: 0.735, 95% CI: 0.590–0.880, *p* = 0.01). Elevated MPV was a significant risk factor for 2-year cardiac mortality (hazard ratio: 2.091, 95% CI: 1.075–4.070, *p* = 0.030) in multivariable Cox regression analysis.
Sivri et al. (2019)	In receiver operating characteristics (ROC) analysis, WMR > 960 predicted a SYNTAX score ≥23 with 80.6% sensitivity and 67.6% specificity (AUC: 0.756; 95% CI: 0.685–0.818; *p* < 0.0001), and a WMR > 1,360 predicted a SYNTAX score ≥33 with 71.4% sensitivity and 93% specificity (AUC: 0.840; 95% CI: 0.777- 0.892; *p* < 0.0001). Higher WMR was associated with a greater SYNTAX score in patients with NSTEMI. WMR may be used to predict the severity of the CAD and to implement risk stratification in patients with NSTEMI.
Demir et al. (2023)	After adjustment for age, sex, eGFR, troponin levels, and the Global Registry of Acute Coronary Events (GRACE) score in Cox regression models, the association of high WMR with the cumulative incidence of MACE was preserved (overall patients (HR = 1.85, 95% CI: 1.1–3.12, *p* = 0.02) and patients with a SYNTAX score <22 (HR = 2.06, 95% CI 1.15–3.67, *p* = 0.01).

NRBC, nucleated red blood cell; MPV, mean platelet volume; NLR, neutrophil to lymphocyte ratio; SII, systemic inflammatory immunologic index; PLR, platelet–lymphocyte ratio; hsCAR, high-sensitivity C-reactive protein–albumin ratio; CAD, coronary artery disease; rSS, residual SYNTAX score; WMR, white blood cell counts to mean platelet volume ratio; P-PCI, primary percutaneous coronary intervention; MACE, major advanced cardiac event; HR, hazard ratio; AUC, area under the curve; NSTEMI, non-ST-elevation myocardial infarction; STEMI, ST-elevation myocardial infarction.

Prospective cohort study by Rostami et al. (37) included the SYNTAX score and the TIMI flow grade before and after primary percutaneous coronary intervention (pPCI), describing the NLR as an independent predictor of postprocedural no reflow, but not a predictor of the SYNTAX score and the preprocedural TIMI flow grade in patients with ST-segment elevation myocardial infarction (STEMI). However, Maleki et al. (20) demonstrated that higher NLR was significantly associated with higher SYNTAX score (*β* = 0.162, *p* = 0.021), and with the same conclusion, Li et al' (22) described that the level of NLR, among other variables, in patients with high SYNTAX score is significantly higher than that in patients with the low SYNTAX score. Zuin et al. (38) demonstrated that the NLR significantly correlated with SYNTAX score with 1-year cardiovascular mortality in patients with ST-segment elevation myocardial infarction (STEMI) or non-ST-segment elevation myocardial infarction (NSTEMI) treated with percutaneous coronary intervention (PCI) within 24 h (OR = 2.85, 95% CI: 1.54–5.26, *p* = 0.001 and OR = 2.57, 95% CI: 1.62–4.07, *p* < 0.0001 for STEMI and NSTEMI, respectively). Kurtul et al. (39) demonstrated that NLR was significantly lower in patients with a low SYNTAX score compared with that in patients with an intermediate or high SYNTAX score (3.7 ± 4–4.6 ± 2 and 7.9 ± 4, *p* < 0.001), and linear regression analysis revealed that NLR (coefficient *β* = 0.380, 95% CI: 1.165–1.917, *p* < 0.001) was significantly associated with the SYNTAX score in patients with NSTEMI.

The residual SYNTAX score (rSS) is used to determine the severity of obstructive coronary atherosclerosis after initial PCI, and in multivariate logistic regression analysis, the NLR was an independent predictor of high rSS (OR = 3.933; 95% CI: 2.419–6.393; *p* < 0.001) (40). Highly sensitive troponin T and NLR were significantly correlated with angiographic severity of acute coronary syndromes (ACS) assessed by SYNTAX score (41). Therefore, many studies have demonstrated the relationship between NLR and the severity of coronary angiographic lesions represented by the SYNTAX score (42–49).

Previous studies have demonstrated the importance of the participation of mean platelet volume (MPV) as a biomarker of coronary atherosclerotic plaque instability and, consequently, in the relationship with angiographic lesions (SYNTAX score). Vogiatzis et al. (29) described that mean platelet volume (MPV) is a primary indicator of platelet activation. It was significantly correlated to SYNTAX score (*r* = 0.658, *p* < 0.001) and was found to be an independent predictor factor of major advanced cardiac event (MACE) with HR = 6.8 (95% CI: 1.46–33.36). Sahin et al. (50) demonstrated that the association with the extent and complexity of coronary artery disease in diabetic patients with ST-elevation myocardial infarction (STEMI) was stronger than that in non-diabetic STEMI patients (*r* = 0.473, *p* < 0.001 vs. *r* = 0.129, *p* = 0.001). A recent study by Abalı et al. (51) described the relationship between MVP and the severity of coronary atherosclerosis in patients with diabetes mellitus, and they found that MPV has a positive correlation with the SYNTAX score. Ekici et al. (52) were quite categorical in clarifying that a positive correlation between MPV and SYNTAX score (*p* < 0.001, *r* = 0.504).

Therefore, numerous studies have explored the relationship between these hematological variables (NLR and MPV) with the SYNTAX score (53–55). However, there is limited research examining the nucleated red blood cells (NRBC) with a biomarker in relation to the SYNTAX score. These biomarkers may have great applicability in clinical practice.

## 5 Discussion

Hematological variables (NRBC, NLR, and MPV), representing the entire spectrum of blood counts in the peripheral circulation, are associated with inflammation and hypoxemia. Coronary angiographic lesions have eminently inflammatory and hematological components, suggesting a likely association between the severity of coronary atherosclerotic plaques (SYNTAX score) and hematological variables. There is substantial evidence in the literature supporting the relationship between mean platelet volume and neutrophil to lymphocyte ratio with coronary angiographic lesions (SYNTAX score), which has not yet been demonstrated with the nucleated red blood cells (NRBC).

These hematological parameters (NRBC, MPV, NLR) have shown potential as valuable tools for in-hospital surveillance of all-cause mortality in patients hospitalized with acute myocardial infarction. However, there is a need for further studies to demonstrate the relationship between these hematological variables and coronary angiographic lesions. In addition to being easy to measure and low-cost, they could serve as useful components of a prognostic scoring system.

## References

[1] Babes EE Zaha DC Tit DM Nechifor AC Bungau S Andronie-Cioara FL Value of hematological and coagulation parameters as prognostic factors in acute coronary syndromes. Diagnostics (Basel). (2021) 11(5):850. 10.3390/diagnostics11050858 34065132 PMC8151317

[2] Monteiro Junior JGM Torres DOC Silva MCFC Príncipe TRN Vasconcelos RB Brito MEC Performance of a hematological scoring system in predicting all-cause mortality in patients with acute myocardial infarction. Int J Cardiovasc Sci. (2020) 33(4):380–8. 10.36660/ijcs.20190094

[3] Monteiro Junior JGM Torres DOC Filho SD. Hematological parameters as prognostic biomarkers in patients with cardiovascular diseases. Curr Cardiol Rev. (2019) 15(4):274–82. 10.2174/1573403X15666190225123544 30799790 PMC6823671

[4] Viana MS Correia VCA Ferreira FM Lacerda YF Bragano GO Fonseca LL Competência prognóstica distinta entre modelo clínico e anatômico em síndromes coronarianas agudas: comparação por tipo de desfecho. Arq Bras de Cardiol. (2020) 115(2):226–8. 10.36660/abc.20190062 PMC8384280 32876188

[5] Libby P Buring JE Badimon L Hansson GK Deanfield J Bittencourt MS. Atherosclerosis. Nat Rev Dis Prim. (2019) 5:56. 10.1038/s41572-019-0106-z 31420554

[6] Kraaijenhof JM Hovingh GK Stroes ES Kroon J. The iterative lipid impact on inflammation in atherosclerosis. Curr Opin Lipidol. (2021) 32(5):286–92. 10.1097/MOL.0000000000000779 34392272 PMC8452331

[7] Chiorescu RM Mocan M Inceu AI Buda AP Blendea D Vlaicu SI. Vulnerable atherosclerotic plaque: is there a molecular signature? Int J Mol Sci. (2022) 23(21):13638. 10.3390/ijms232113638 36362423 PMC9656166

[8] Tosun H Kamışlı S Tecellioğlu M Alan S Tecellioğlu FS Öztanır MN Red and white thrombus characteristics in patients undergoing carotid endarterectomy. J Stroke Cerebrovasc Dis. (2021) 30(2):105451. 10.1016/j.jstrokecerebrovasdis.2020.105451 33278805

[9] Asada Y Yamashita A Sato Y Hatakeyama K. Pathophysiology of atherothrombosis: mechanisms of thrombus formation on disrupted atherosclerotic plaques. Pathol Int. (2020) 70(6):309–22. 10.1111/pin.12921 32166823 PMC7317428

[10] Guyton AC Hall JE. Textbook of Medical Physiology. 11th ed. Rio de Janeiro: Elsevier Editora Ltda (2006). ISBN 978-85-352-1641-7; 32: 419-28; 33: 429-38; 36: 457- 68.

[11] Monteiro Junior JGM Sobral Filho DC. Potential role of hematological parameters in patients with acute myocardial infarction: viewpoint. Int J Cardiovasc Sci. (2020) 33(5):586–8. 10.36660/ijcs.20200108

[12] Stachon A Sebbers E Holland-Letz T Kempf R Herinf S Krieg M. Nucleated red blood cells in the blood of medical intensive care patients indicate increased mortality risk: a prospective cohort study. Crit Care. (2007) 11(3):R62. 10.1186/cc5932 17550592 PMC2206423

[13] Kuert S Holland-Letz T Friese J Stachon A. Association of nucleated red blood cells in blood and arterial oxygen partial tension. Clin Chem Lab Med. (2011) 49(2):257–63. 10.1515/CCLM.2011.041 21118046

[14] Desai S Jones SL Turner KL Hall J Moore LJ. Nucleated red blood cells are associated with a higher mortality rate in patients with surgical sepsis. Surg Infect (Larchmt). (2012) 13(6):360–5. 10.1089/sur.2011.089 23237100

[15] Yalcinkaya E Yuksel UC Celik M Kabul HK Barcin C Gokoglan Y Relationship between the neutrophil/lymphocyte ratio and electrocardiographic ischemia grade in the STEMI. Arq Bras Cardiol. (2015) 104(2):112–9. 10.5935/abc.20140179 25424159 PMC4375654

[16] Núñez J Núñez E Bodí V Sanchis J Mainar L Miñana G Low lymphocyte count in acute phase of ST-segment elevation myocardial infarction predicts long-term recurrent myocardial infarction. Coronary Artery Dis. (2010) 21:1–7. 10.1097/mca.0b013e328332ee15 20050312

[17] Kaya H Ertaş F İslamoğlu Y Kaya Z Atılgan ZA Çil H Association between neutrophil to lymphocyte ratio and severity of coronary artery disease. Clin Appl Thromb Hemost. (2014) 20(1):50–4. 10.1177/1076029613499821 22790659

[18] Verdoia M Barbieri L Di Giovine G Marino P Suryapranata H De Luca G. Neutrophil to lymphocyte ratio and the extent of coronary artery disease: results from a large cohort study. Angiology. (2016) 67(1):75–82. 10.1177/0003319715577529 25818102

[19] Azab B Zaher M Weiserbs KF Torbey E Lacossiere K Gaddam S Usefulness of neutrophil to lymphocyte ratio in predicting short and long-term mortality after non-ST elevation myocardial infarction. Am J Cardiol. (2010) 106(4):470–6. 10.1016/j.amjcard.2010.03.06 20691303

[20] Maleki M Tajlil A Separham A Sohrabi B Pourafkari L Roshanravan N Association of neutrophil to lymphocyte ratio (NLR) with angiographic SYNTAX score in patients with non-ST-segment elevation acute coronary syndrome (NSTE-ACS). J Cardiovasc Thorac Res. (2021) 13(3):216–21. 10.34172/jcvtr.2021.40 34630969 PMC8493237

[21] Shahsanaei F Abbaszadeh S Behrooj S Rahimi Petrudi N Ramezani B. The value of neutrophil-to-lymphocyte ratio in predicting severity of coronary involvement and long-term outcome of percutaneous coronary intervention in patients with acute coronary syndrome: a systematic review and meta-analysis. Egypt Heart J. (2024) 76(1):39. 10.1186/s43044-024-00469-3 38546902 PMC10978563

[22] Li S Chen H Zhou L Cui H Liang S Li H. Neutrophil-to-lymphocyte ratio predicts coronary artery lesion severity and long-term cardiovascular mortality in patients with unstable angina pectoris. Acta Cardiol. (2022) 77(8):708–15. 10.1080/00015385.2021.1963564 35969267

[23] Kaplangoray M Toprak K Aslan R Deveci E Gunes A Ardahanli İ. High CRP-albumin ratio is associated with high thrombus burden in patients with newly diagnosed STEMI. Medicine (Baltimore). (2023) 102(41):e35363. 10.1097/MD.0000000000035363 37832116 PMC10578711

[24] Yu M Wang Y Yang S Mei J Liu Z Zhang L Elucidating the relationship between neutrophil-lymphocyte ratio and plaque composition in patients with drug-eluting stent restenosis by virtual histology-intravascular ultrasound. J Cardiovasc Dev Dis. (2024) 11(7):211. 10.3390/jcdd11070211 39057631 PMC11276828

[25] Wendland AE Farias MG Manfroi WC. Volume plaquetário médio e doenca cardiovascular. J Bras Patol Med Lab. (2009) 45(5):371–8. 10.1590/S1676-24442009000500005

[26] Uysal HB Dağlı B Akgüllü C Avcil M Zencir C Ayhan M Blood count parameters can predict the severity of coronary artery disease. Korean J Intern Med. (2016) 31:1093–100. 10.3904/kjim.2015.199 27052265 PMC5094927

[27] Murat SN Duran M Kalay N Gunebakmaz O Akpek M Doger C Relation between mean platelet volume and severity of atherosclerosis in patients with acute coronary syndromes. Angiology. (2013) 64(2):131–6. 10.1177/0003319711436247 22334878

[28] Klovaite J Benn M Yazdanyar S Nordestgaard BG. High platelet volume and increased risk of myocardial infarction: 39,531 participants from the general population. J Thromb Hemost. (2011) 9(1):49–56. 10.1111/j.1538-7836.2010.04110.x 20942852

[29] Vogiatzis I Samaras A Grigoriadis S Sdogkos E Koutsampasopoulos K Bostanitis I. The mean platelet volume in the prognosis of coronary artery disease severity and risk stratification of acute coronary syndromes. Med Arch. (2019) 73(2):76–80. 10.5455/medarh.2019.73.76-80 31391691 PMC6643353

[30] Vukicevic P Klisic A Neskovic V Babic L Mikic A Bogavac-Stanojevic N New markers of platelet activation and reactivity and oxidative stress parameters in patients undergoing coronary artery bypass grafting. Oxid Med Cell Longev. (2021) 2021:8915253. 10.1155/2021/8915253 34257821 PMC8257340

[31] Serruys PW Morice MC Kappetein AP Colombo A Holmes DR Mack MJ Percutaneous coronary intervention versus coronary artery bypass grafting for severe coronary artery disease. N Engl J Med. (2009) 360(10):961–72. 10.1056/NEJMoa0804626 19228612

[32] Chakrabarti AK Gibson CM. The SYNTAX score: usefulness, limitations, and future directions. J Invasive Cardiol. (2011) 23(12):511–2. https://www.hmpgloballearningnetwork.com/site/jic/articles/syntax-score-usefulness-limitations-and-future-directions 22147398

[33] Wood S. Syntax Tool Unvieled at EuroPCR: Now the Trick is to Use It. Available online at: https://www.theheart.org/article/973297.do (May 22, 2009).

[34] Bundhun PK Sookharee Y Bholee A Huang F. Application of the SYNTAX score in interventional cardiology: a systematic review and meta-analysis. Medicine (Baltimore). (2017) 96(28):e7410. 10.1097/MD.0000000000007410 28700477 PMC5515749

[35] Özmen M Arikan E Ozel F Ardahanlı I. Triglyceride-glucose index: evaluation as a potential new risk marker for SYNTAX score in acute coronary syndrome. Int J Cardiovasc Sci. (2024) 37:e20240096. 10.36660/ijcs.20240096

[36] Xu B Généreux P Yang Y Leon MB Xu L Qiao S Validation and comparison of the long-term prognostic capability of the SYNTAX score-II among 1,528 consecutive patients who underwent left main percutaneous coronary intervention. JACC Cardiovasc Interv. (2014) 7(10):1128–37. 10.1016/j.jcin.2014.05.018 25240551

[37] Rostami A Tajlil A Separham A Sohrabi B Pourafkari L Roshanravan N Association between neutrophil-to-lymphocyte ratio and the systemic inflammatory immunologic index and the angiographic SYNTAX score and the TIMI flow grade in acute STEMI: a cohort study. J Tehran Heart Cent. (2021) 16(4):147–55. 10.18502/jthc.v16i4.8600 35935551 PMC9308885

[38] Zuin M Rigatelli G Picariello C dell'Avvocata F Marcantoni L Pastore G Correlation and prognostic role of neutrophil to lymphocyte ratio and SYNTAX score in patients with acute myocardial infarction treated with percutaneous coronary intervention: a six-year experience. Cardiovasc Revasc Med. (2017) 18(8):565–71. 10.1016/j.carrev.2017.05.007 28529092

[39] Kurtul S Sarli B Baktir AO Demirbas M Saglam H Doğan Y Neutrophil to lymphocyte ratio predicts SYNTAX score in patients with non-ST segment elevation myocardial infarction. Int Heart J. (2015) 56(1):18–21. 10.1536/ihj.14-175 25742940

[40] Kahraman S Agus HZ Avci Y Serbest NG Guner A Erturk M. The neutrophil to lymphocyte ratio (NLR) is associated with residual syntax score in patients with ST-segment elevation myocardial infarction. Angiology. (2021) 72(2):166–73. 10.1177/0003319720958556 32945176

[41] Altun B Turkon H Tasolar H Beggı H Altun M Temız A The relationship between high-sensitive troponin T, neutrophil lymphocyte ratio and SYNTAX score. Scand J Clin Lab Invest. (2014) 74(2):108–15. 10.3109/00365513.2013.860619 24304492

[42] Pan Q Zhang W Li X Chen Z Yang Y Wang G. Sex difference in the association between neutrophil to lymphocyte ratio and severity of coronary artery disease. Angiology. (2022) 73(5):470–7. 10.1177/00033197211070884 35129378

[43] Demir K Avci A Altunkeser BB Yilmaz A Keles F Ersecgin A. The relation between neutrophil-to-lymphocyte ratio and coronary chronic total occlusions. BMC Cardiovasc Disord. (2014) 14:130. 10.1186/1471-2261-14-130 25260530 PMC4195893

[44] Soylu K Gedikli Ö Dagasan G Aydin E Aksan G Nar G Neutrophil-to-lymphocyte ratio predicts coronary artery lesion complexity and mortality after non-ST-segment elevation acute coronary syndrome. Rev Port Cardiol. (2015) 34(7-8):465–71. 10.1016/j.repc.2015.01.013 26164277

[45] Kaya A Kurt M Tanboga IH Işık T Günaydın ZY Kaya Y Relation of neutrophil to lymphocyte ratio with the presence and severity of stable coronary artery disease. Clin Appl Thromb Hemost. (2014) 20(5):473–7. 10.1177/1076029612473517 23344996

[46] Sönmez O Ertaş G Bacaksız A Tasal A Erdoğan E Asoğlu E Relation of neutrophil-to-lymphocyte ratio with the presence and complexity of coronary artery disease: an observational study. Anadolu Kardiyol Derg. (2013) 13(7):662–7. 10.5152/akd.2013.188 23912788

[47] Sari I Sunbul M Mammadov C Durmus E Bozbay M Kivrak T Relation of neutrophil-to-lymphocyte and platelet-to-lymphocyte ratio with coronary artery disease severity in patients undergoing coronary angiography. Kardiol Pol. (2015) 73(12):1310–6. 10.5603/KP.a2015.0098 25987404

[48] Sahin DY Elbasan Z Gür M Yildiz A Akpinar O Icen YK Neutrophil to lymphocyte ratio is associated with the severity of coronary artery disease in patients with ST-segment elevation myocardial infarction. Angiology. (2013) 64(6):423–9. 10.1177/0003319712453305 22802534

[49] Tanındı A Erkan AF Ekici B Alhan A Töre HF. Neutrophil to lymphocyte ratio is associated with more extensive, severe and complex coronary artery disease and impaired myocardial perfusion. Turk Kardiyol Dern Ars. (2014) 42(2):125–30. 10.5543/tkda.2014.18949 24643143

[50] Sahin DY Gür M Elbasan Z Özdoğru I Uysal OK Kivrak A Mean platelet volume and extent and complexity of coronary artery disease in diabetic and nondiabetic patients with ST elevation myocardial infarction. Angiology. (2013) 64(7):505–11. 10.1177/0003319712460423 23028178

[51] Abalı G Akpınar O Söylemez N. Correlation of the coronary severity scores and mean platelet volume in diabetes mellitus. Adv Ther. (2014) 31(1):140–8. 10.1007/s12325-013-0081-9 24318519

[52] Ekici B Erkan AF Alhan A Sayın I Aylı M Töre HF. Is mean platelet volume associated with the angiographic severity of coronary artery disease? Kardiol Pol. (2013) 71(8):832–8. 10.5603/KP.2013.0195 24049023

[53] Sivri S Sokmen E Celik M Ozbek SC Yildirim A Boduroglu Y. Usefulness of white blood cell count to mean platelet volume ratio in the prediction of SYNTAX score in patients with non-ST elevation myocardial infarction. Pak J Med Sci. (2019) 35(3):824–9. 10.12669/pjms.35.3.1017 31258602 PMC6572941

[54] Emre AR Yasar KA Atakan Y Orhan C Murathan K. Relationship between white blood count to mean platelet volume ratio and clinical outcomes and severity of coronary artery disease in patients undergoing primary percutaneous coronary intervention. Cardiovasc Ther. (2020) 2020:9625181. 10.1155/2020/9625181 32934665 PMC7482024

[55] Şenöz O Emren SV Erseçgin A Yapan Emren Z Gül İ. Platelet-Lymphocyte ratio is a predictor for the development of no-reflow phenomenon in patients with ST-segment elevation myocardial infarction after thrombus aspiration. J Clin Lab Anal. (2021) 35(6):e23795. 10.1002/jcla.23795 33945171 PMC8183944

